# The Odyssey of Entropy: Cryptography

**DOI:** 10.3390/e24020266

**Published:** 2022-02-12

**Authors:** Behrouz Zolfaghari, Khodakhast Bibak, Takeshi Koshiba

**Affiliations:** 1 Cyber Science Lab, School of Computer Science, University of Guelph, Guelph, ON N1G 2W1, Canada; behrouz@cybersciencelab.org; 2 Department of Computer Science and Software Engineering, Miami University, Oxford, OH 45056, USA; 3 Department of Mathematics, Faculty of Education and Integrated Arts and Sciences, Waseda University, Tokyo 169-8050, Japan; tkoshiba@waseda.jp

**Keywords:** entropy, cryptography, security, information theory, trend analysis

## Abstract

After being introduced by Shannon as a measure of disorder and unavailable information, the notion of entropy has found its applications in a broad range of scientific disciplines. In this paper, we present a systematic review on the applications of entropy and related information-theoretical concepts in the design, implementation and evaluation of cryptographic schemes, algorithms, devices and systems. Moreover, we study existing trends, and establish a roadmap for future research in these areas.

## 1. Introduction

In thermodynamics and statistical mechanics, the internal disorder of a system in a given macroscopic state is stated as a logarithmic function of the number Ω of possible microscopic system configurations as follows,

(1) $$S=k_{B}\ln\Omega .$$

In Equation (1), *S* is referred to as the *entropy* of the system, and $k_{B}$ is called the *Boltzmann constant*. Under the equiprobability assumption, it is obvious that Ω is an exponential function of the number of particles that can randomly move within the system. In other words, lnΩ is proportional to the number of random particles inside the system, which is a measure of randomness. The Boltzmann constant converts this number to the total uncontrolled kinetic energy of the random particles. On the other hand, the number of randomly moving particles inside a system can be considered to be representative of the amount of information that is needed to define the exact state of a system given its macroscopic state.

According to the above discussions, the thermodynamic concept of entropy connects uncontrolled energy inside a system to disorder, randomness and unavailable information. However, the term entropy has found its applications with different notions in a variety of scientific disciplines, ranging from cosmology and meteorology to economics, biology, medicine and sociology. In particular, Shannon paved the way ahead of this concept via information theory into communication theory and related fields, such as cryptography.

Information entropy (information theoretic entropy) was first introduced by Shannon in 1948 [1,2]. It can be assigned to a random variable as the average level of *self-information* in each possible event of the variable, which shows the inherent level of uncertainty or surprise in the event. In Shannon’s theory, for a random variable *X*, the self-information $I_{X}$ of an event $x_{i}$ with probability $P_{X}\left(x_{i}\right)$ is defined as

(2) $$I_{X}\left(x_{i}\right)=-\log_{b}P_{X}\left(x_{i}\right).$$

In Equation (2), the base *b* determines the unit of information. In particular, if b=2, $I_{X}\left(x_{i}\right)$ is calculated in bits. Moreover, the entropy *H* of *X* is defined as

(3) $$H\left(X\right)=E\left[I_{X}\right]=\sum_{i}P_{X}\left(x_{i}\right)I_{X}\left(x_{i}\right)=-\sum_{i}P_{X}\left(x_{i}\right)\log_{b}P_{X}\left(x_{i}\right).$$

In Equation (3), $E\left[I_{X}\right]$ is the mathematical expectation of $I_{X}$. Von Neumann suggested the name “entropy” for the concept introduced by Shannon because of its similarity to thermodynamic entropy in notion as well as related equations. In fact, information-theoretic entropy is used as a measure of randomness, disorder and unavailable information, like the case of thermodynamic entropy. Shannon discussed the role of entropy and related concepts in the modeling of cryptosystems. Further, he introduced the notion of a perfectly secure cryptosystem on the basis of entropy.

Some different notions of information entropy were introduced by other researchers before [3] and after [4] Shannon. In the rest of this paper, the term “entropy” refers to information-theoretic entropy, unless we clearly specify thermodynamic entropy. Entropy has found its applications in a variety of scientific and technological areas [5,6,7]. Many research reports have addressed the role of entropy in the design, implementation and analysis of cryptosystems as well as cryptographic applications and environments. Several survey reports have reviewed the applications of entropy in a variety of areas, such as economics [8], image processing [9], discrete mathematics [10], signal processing [11], etc.

There are some research reports that establish connections between the notion of entropy and elements or requirements of cryptosystems. For example, the role of entropy in the calculation of the lower bounds on key size as well as the relation between entropy and perfect secrecy were studied by Maurer [12]. Moreover, a quick introduction to some entropy-related notions in cryptosystems was presented by Reyzby [13]. The relation between entropy and true randomness as well as key unpredictability was studied by Vassilev and Hall [14]. However, to the best of our knowledge, there is no up-to-date, systematic and comprehensive review on the applications of entropy in cryptography and related areas. Thus, a systematic and comprehensive survey in this area can help researchers by shedding light on hot topics as well as the ones that need more research focus.

**Figure 1:**
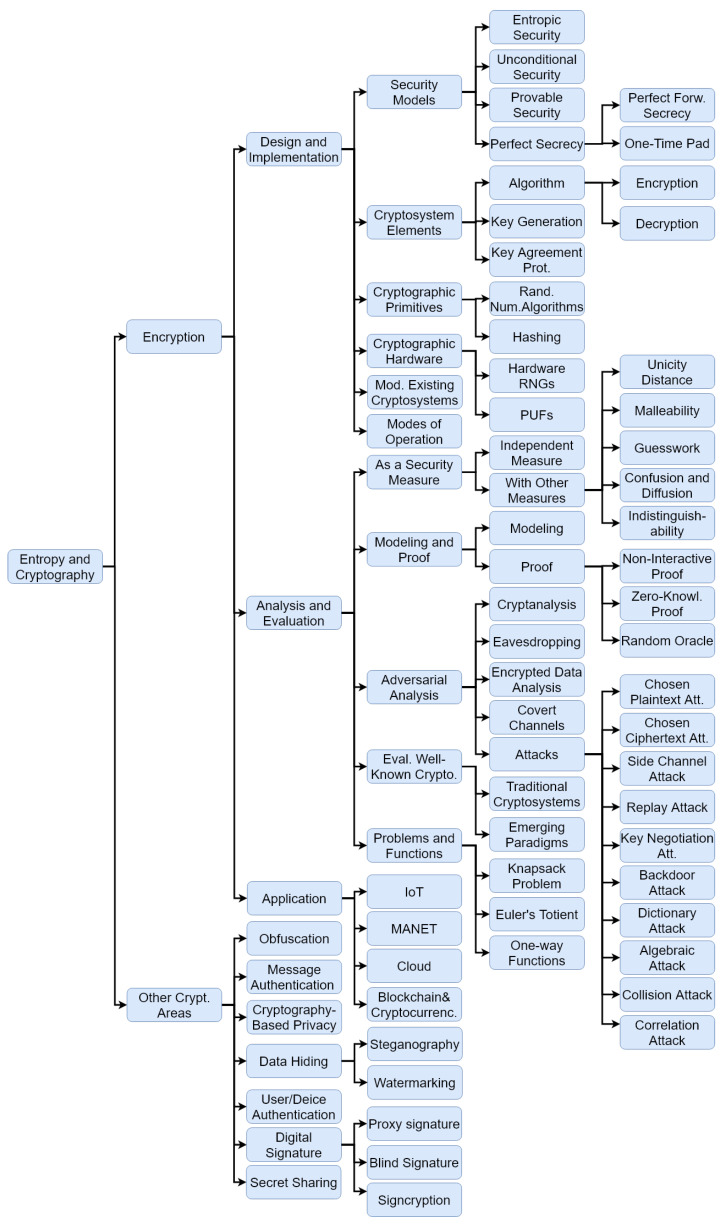
The classification of cryptographic concepts with entropy-related aspects.

In this paper, we review and classify the roles and applications of entropy and related concepts in different branches of cryptography. We analyze the existing research trends in this area, and establish a future roadmap for further research in this area. In this survey, we divide the papers into those focused on the applications of entropy in encryption and those focused on other related cryptographic concepts, such as obfuscation, watermarking, etc. We classify encryption-related research works on the basis of the phases in a typical life cycle of a cryptosystem, namely, design, implementation, evaluation and application as shown in Figure 1.

The rest of this paper is organized as follows. Some entropy measures and related concepts are studied in Section 2. Section 3 studies the applications of entropy in encryption, and Section 4 reviews the applications of entropy in other cryptographic areas. Section 5 analyses the current trends, and presents a roadmap for future research on the applications of entropy in cryptography.

## 2. Entropy Measures and Related Concepts

In the next sections of this paper, we review the roles of different entropy measures and related concepts in research on cryptography. Some of the most important measures and concepts are briefly introduced in this section.

### 2.1. Entropy Measures

In addition to Shannon entropy, some other measures for entropy have been introduced by different researchers. For example, for a random variable *X* with events $x_{i}=i$ (i∈{1,2,…,n}) of probability $P_{X}\left(x_{i}\right)$, Rényi entropy $H_{\alpha }^{\left(R\right)}$ of order $\alpha \in R^{+}\backslash \left\{1\right\}$ is defined as [4],

(4) $$H_{\alpha }^{\left(R\right)}\left(X\right)=\frac{1}{1-\alpha }\log_{2}\sum_{i=1}^{n}{\left(P_{X}\left(x_{i}\right)\right)}^{\alpha }.$$

It is well known that $\lim_{\alpha \to 1}H_{\alpha }^{\left(R\right)}\left(X\right)=H\left(X\right)$, where $H\left(X\right)$ is the Shannon entropy given by Equation (3). Moreover, $H_{0}^{\left(R\right)}\left(X\right)=H^{\left(H\right)}\left(X\right)$, where $H^{\left(H\right)}\left(X\right)$ is the Hartley entropy (sometimes called max entropy) defined as

(5) $$H^{\left(H\right)}\left(X\right)=\max_{\alpha }H_{\alpha }^{\left(R\right)}\left(X\right)\log_{2}n=\log_{2}\left|X\right|.$$

*Collision entropy*, defined below, is used by many researchers in cryptography,

(6) $$H^{\left(C\right)}\left(X\right)=H_{2}^{\left(R\right)}\left(X\right)=-\log_{2}\sum_{i=1}^{n}{\left(P_{X}\left(x_{i}\right)\right)}^{2}.$$

The security of cryptosystems is measured by the expectation of certain function, which is called *perfect expectation* (in the ideal model, where perfect randomness is available) or *weak expectation* (in the real model, where perfect randomness is not available). Yao and Li [15] used Rényi entropy to derive some results and inequalities, which show that weak expectation is not much worse than perfect expectation. For a cryptosystem C, let resource R be a tuple containing the values of all efficiency measures, such as running time, circuit size, the number of oracle queries, etc. For a secret key $k\in {\left\{0,1\right\}}^{m}$, let f(k) be the advantage of adversary A conditioned on *k*. In addition, let $U_{m}$ be the uniform distribution over ${\left\{0,1\right\}}^{m}$. Then C is (R,ε)-secure if for any adversary A with resource R, the expectation of $f\left(U_{m}\right)$ (called *perfect expectation*) is upper bounded by ε [16]. When the key is sampled from some non-uniform distribution *W*, the resulting security is the expectation of f(W) (called *weak expectation, Weak-Expect-Conf001*). The result of Yao and Li [15] was motivated by the fact that while cryptographic schemes ideally assume highly random secret keys, true random number generation is so costly and sometimes not feasible in the real world, which causes weak expectation to be substituted for the ideal perfect expectation. As another example, Rényi entropy was used by Boztas [17] in order to analyze the success parameters of guess attacks.

The relation between the results of evaluating a cryptosystem using Shannon entropy and collision entropy was studied by Skorski [18], and the worst possible collision entropy for random variables with a given Shannon entropy was calculated.

Another well-known entropy measure is *min entropy*, $H^{\left(M\right)}$, which is calculated as

(7) $$H^{\left(M\right)}\left(X\right)=\min_{\alpha }H_{\alpha }^{\left(R\right)}\left(X\right)=\lim_{\alpha \to \infty }H_{\alpha }^{\left(R\right)}\left(X\right)=-\log_{2}\left(\max_{i}P_{X}\left(x_{i}\right)\right).$$

Min entropy has been used in studies related to cryptographic primitives, such as hash functions [19,20], in developing cryptographic hardware such as PUFs (physically unclonable functions) [21], and in authentication [22,23] and secret sharing [24].

For a probability distribution $P=\{p_{1},p_{2},\ldots ,p_{n}\}$ and $R\in R^{+}\backslash \left\{1\right\}$, *R-norm entropy* $H_{n}^{\left(R\right)}\left(P\right)$ is defined as [25],

(8) $$H_{n}^{\left(R\right)}\left(P\right)=\frac{R}{1-R}\left(1-{\left(\sum_{i=1}^{n}{\left(p_{i}\right)}^{R}\right)}^{\frac{1}{R}}\right).$$

*R*-norm entropy is used in fuzzy probability spaces and related areas [26]. Kumar and Choudhary [27] considered Shannon entropy as a special case of *R*-norm entropy when parameter *R* in Equation (8) approaches unity. They defined conditional *R*-norm entropy as well as *R*-norm mutual information, and used the defined concepts to generalize the notion of random cipher introduced by Shannon.

For a continuous random variable *X* with a probability density function *f* whose support is set χ, the *differential entropy* h(X) is defined as follows [1,2]:

(9) $$h\left(X\right)=-\int_{\chi }f\left(x\right)\log_{2}f(x).$$

Differential entropy has been suggested by some researchers as a measure of security. For example, it was used by Biryukova et al. [28] for cryptanalysis of the well-known IDEA block cipher.

### 2.2. Related Concepts

In addition to entropy measures, there are some related concepts that have played significant roles in research on cryptography. As an example, we can mention relative entropy, conditional entropy and mutual information; the latter is obtained using conditional entropy. For discrete probability distributions *P* and *Q* defined on the probability space S, the *relative entropy* (i.e., the *Kullback–Leibler (KL) divergence*) from *Q* to *P* is defined as [29]

(10) $$D_{\mathrm{KL}}\left(P\|Q\right)=\sum_{x\in S}P\left(x\right)\log\left(\frac{P(x)}{Q(x)}\right).$$

Consider two random variables *X* and *Y* with outcomes $x_{i}$ and $y_{i}$. The *conditional entropy* H(Y|X) is defined as

$$H\left(Y|X\right)$$

(11) $$\begin{array}{cc} = & -\sum_{x_{i}}\sum_{y_{i}}\left(P\left(x_{i},y_{i}\right)\log_{2}P\left(y_{i}|x_{i}\right)\right) \end{array}$$

(12) $$\begin{array}{cc} = & -\sum_{x_{i}}P_{X}\left(x_{i}\right)\sum_{y_{i}}\left(P\left(y_{i}|x_{i}\right)\log_{2}P\left(y_{i}|x_{i}\right)\right). \end{array}$$

$H\left(Y|X\right)$ represents the amount of uncertainty in *Y* given the value of *X*. *Mutual information* between *X* and *Y* is calculated as

(13) $$I\left(X;Y\right)=H\left(X\right)-H\left(X|Y\right)=H\left(Y\right)-H\left(Y|X\right).$$

$I\left(X;Y\right)$ quantifies the amount of information (in bits) obtained about *X* via observing *Y* or vice versa. Moreover, the *joint entropy* H(X,Y) is calculated as

(14) $$H\left(X,Y\right)=I\left(X;Y\right)+H\left(X|Y\right)+H\left(Y|X\right).$$

Moreover, for a distribution $P_{XY}$ and 0<ε<1, the *inf-spectral entropy* $H_{s}^{\varepsilon }\left(P\right)$ is defined as

(15) $$\begin{array}{c} H_{s}^{\varepsilon }\left(P\right)=\sup\left\{r\;|\;P_{XY}\left\{-\log P_{XY}\left(x,y\right)\leq r\right\}\leq \varepsilon \right\}. \end{array}$$

A sequence of jointly distributed random variables $\left(X_{1},X_{2},\ldots ,X_{m}\right)$ has *next-block pseudoentropy* of at least *e* if and only if there exist random variables $\left(Y_{1},Y_{2},\ldots ,Y_{m}\right)$ jointly distributed with $\left(X_{1},X_{2},\ldots ,X_{m}\right)$ such that, for every i∈{1,2,…,m}, $\left(X_{1},\ldots ,X_{i-1},X_{i}\right)$ is computationally indistinguishable from $\left(X_{1},\ldots ,X_{i-1},Y_{i}\right)$ and

$$\sum_{i=1}^{m}H\left(Y_{i}\;|\;X_{1},X_{2},\ldots ,X_{i-1}\right)\geq e.$$

Further, the *conditional Rényi entropy* of order α is calculated as

(16) $$H_{\alpha }^{\left(R\right)}\left(X|Y\right)=\frac{1}{1-\alpha }\max_{y_{i}}\left(\log_{2}\sum_{x_{i}}{\left(P(x_{i}|y_{i})\right)}^{\alpha }\right).$$

Mutual information has played significant roles in several research works in the area of cryptology. For example, Rastegin [30] used it in quantum cryptography in order to measure the amount of information gained by an eavesdropper in each individual attack session. As another example, one can mention the research reported by Gierlichs et al. [31], in which mutual information was used for modeling the information leaked from an embedded device containing a secret key during a side channel attack. Conditional Rényi entropy was used by Iwamoto and Shikata [32] in developing generalizations for Shannon’s secure encryption theorem.

In addition to statistical distributions, entropy can be calculated over different structures, such as graphs. For example, for an undirected graph $G\left(V,E\right)$, the *graph entropy* is defined as [33]

(17) $$\begin{array}{c} H\left(G\right)=\min_{X,Y}I(X;Y), \end{array}$$

where *X* is uniformly distributed over *V*, and *Y* ranges over $I_{S}\left(G\right)$ defined as

(18) $$\begin{array}{c} I_{S}\left(G\right)=\left\{S|S\subset G,X\in S,\forall s_{1},s_{2}\in S:\left(s_{1},s_{1}\right)\notin E\right\}. \end{array}$$

**Figure 2:**
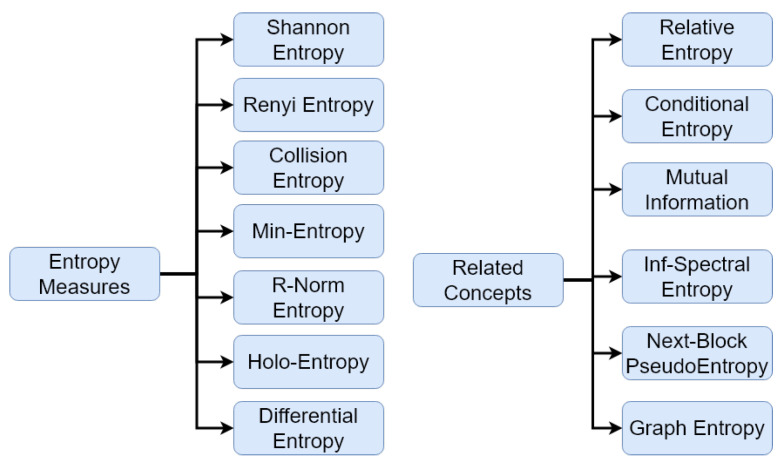
Entropy measures and related concepts used in cryptography.

Figure 2 shows the entropy measures and related concepts that have been used by researchers in the area of cryptography.

## 3. Entropy and Encryption

This section reviews the role of entropy in research on the design, implementation, evaluation and applications of encryption modules and systems.

### 3.1. Modeling, Design and Implementation

In this section, we first study entropy-based security models. Afterwards, we review research works focusing on the role of entropy in the design and implementation of cryptosystem elements, cryptographic primitives and cryptographic hardware. In the next step, we study some entropy-aware modifications to existing cryptosystems. Lastly, we discuss the role of entropy in research on modes of operation, which are considered important implementation aspects of cryptosystems.

#### 3.1.1. Security Models Related to Entropy

In the following, we discuss security models that can be analyzed using entropy, and we review some related research.

- **Entropic Security:** Entropic security is a relaxed version of semantic security. In *semantic security*, only negligible information about the plaintext can be extractable (in any feasible way) from the ciphertext. More specifically, suppose that a probabilistic polynomial time algorithm (PPTA) knows the ciphertext *c* generated from a message *m* (regardless of the related distribution) and the length of *m*. The algorithm should still be unable to extract any partial information regarding *m* with a probability that is non-negligibly larger than all other PPTAs that know only the length of *m* (and not *c*).
  In *entropic security*, the cryptosystem needs to guarantee that the entropy of the message space is high from the point of view of the adversary [34]. A few research reports have worked on entropic security for high-entropy plaintexts [35]. Moreover, this model was used in honey encryption [36]. In honey encryption, decrypting the ciphertext using an incorrect key (guessed by the adversary) leads to a meaningful, but incorrect plaintext, which fools the adversary.
- **Unconditional Security:** A cryptosystem is said to have *unconditional security* (also called *information-theoretic security*) if the system is secure against adversaries with unlimited computational power, resources, memory space, and time. Information-theoretic methods and techniques have been utilized in studying unconditionally secure cryptosystems [37]. Some researchers have focused on this security model. For example, Renner and Wolf [38] investigated the possibility of asymmetric unconditional security, which corresponds to asymmetric-key cryptography in the computational security model.
- **Provable Security:** Some researchers have used entropy in provable security. For example, Kim et al. [39] argued that the assumption of uniform key distribution, which is made in traditional provable security, is far from reality. They modeled realistic key distributions by entropy sources. As another example, it was shown by Ruana [40] that the explicit authenticated key agreement protocol presented by Zheng [41] is vulnerable to impersonation attack due to the low entropy of the keys.
- **Perfect Secrecy:** Perfect secrecy (defined by Shannon) guarantees that H(P|C)=H(P), where *P* is the set of possible values for the plaintext and *C* denotes the set of possible values for the ciphertext. Put alternatively, a cryptosystem is perfectly secure if the adversary is unable to make any guesses about the plaintext, even in the case of full access to the channel (and, consequently, to the ciphertext). Several research works have focused on perfect secrecy. Gersho [42] argued that message quality degradation is inevitable for a perfectly secure cryptosystem that encrypts an analog message using a digital key with a finite size. He designed a perfectly secure analog signal encryption scheme that keeps the bandwidth of the encrypted signal from growing above that of the original analog signal without altering the key size or increasing the quality degradation incurred on the decrypted signal. The notion of “finite-state encryptability” for an individual plaintext sequence was introduced by Merhav [43] as the minimum asymptotic key rate required to guarantee perfect secrecy for that sequence. He demonstrated that the finite-state encryptability is equal to the finite-state compressibility (defined by Ziv and Lempel [44]) for every individual sequence. Perfect secrecy in radio signal encryption using DFT (discrete Fourier transform) was studied by Bi et al. [45]. They proved perfect secrecy to be asymptotically achievable for any baseband signaling method, provided that the signal block length approaches infinity. It is well known that the only real-world implementation of perfect secrecy (in its pure notion) is one-time pad (OTP), wherein the key is at least as long as the plaintext and needs to be updated with each new plaintext. However, some variants of perfect secrecy have received research focus. In the following, we review some well-studied variants of perfect secrecy as well as some related research works.
  - **Perfect Forward Secrecy:**
    Perfect forward secrecy depends on frequent changes in the encryption/decryption key (e.g., with each call or each message in a conversation, or each web page reload) in order to prevent the cryptosystem from being broken if a key is compromised. Several researchers have proposed encryption systems providing perfect forward secrecy to be used in different communication systems. For example, two email protocols with perfect forward secrecy were proposed by Sun et al. [46]. However, some flaws in the reasoning presented by Sun et al. [46] were reported by Dent [47], and two new robust email protocols with guaranteed perfect forward secrecy were introduced by Ziv and Lempel [48]. Later on, a method for cryptanalysis of the protocols proposed by Ziv and Lempel [48] was presented by Yoon and Yoo [49].
    In recent years, perfect forward secrecy has been considered in several other areas. For example, a lightweight transport layer security protocol with perfect secrecy was proposed by Pengkun et al. [50]. As another example, perfect secrecy is guaranteed to be provided by a high-performance key agreement protocol proposed by Yang et al. [51].
  - **One-Time Pad (OTP):**
    OTP is the only perfectly secure cryptographic scheme used in real-world applications. Although some researchers believe that OTP is more of a key safeguarding scheme than a cryptosystem [52], a vast number of research works have considered OTP as (part of) the security solution in a broad spectrum of applications. The feasibility of perfectly secure cryptography using imperfect random sources was studied by Dodis and Spencer [53]. Liu et al. [54] proposed an OTP cryptosystem in which the receiver does not need the OTP to decrypt the ciphertext, while the OTP fully affects the plaintext from the adversaries point of view. The application of OTP in scenarios where the receiver may not be trustworthy was studied by Matt and Maurer [55].
    An OTP-based cryptosystem was proposed by Büsching and Wolf [56] for BANs (body area networks), wherein messages are short, and large volumes of NVM (non-volatile memory) are available. This cryptosystem stores pre-calculated OTPs in the NVM for future use. Moreover, OTPs have been used in a spectrum of environments, such as multi-user one-hop wireless networks [57], IMDs (implantable medical devices) [58], UAVs (ynmanned aerial vehicles) [59], medical images [60], mobile instant messaging [61], coded networks [62] and credit cards [63]. OTP has been also used in quantum computing, especially in QKD (quantum key distribution) [64,65,66,67,68,69,70].

#### 3.1.2. Cryptosystem Elements

The security of a cryptosystem depends on the security of three main elements: the encryption and decryption algorithms, the key generation and management module, and the key agreement or exchange protocol [71]. In the following, we review the role of entropy and related concepts in the design and implementation of each of the aforementioned elements.

- **Encryption and Decryption Algorithms:** Entropy has played a role in several research reports focusing on the design of encryption and decryption algorithms. Some of these research works are discussed below.
  - **Encryption:**
    The role of image block entropy in image encryption was studied by researchers [72] just like the case of image steganography (reviewed in Section 4.3.1). A multimedia encryption scheme based on entropy coding with low computational overhead was proposed by Xie and Kuo [73]. A method for encrypting entropy-coded compressed video streams without the need for decoding was introduced by Almasalha et al. [74]. Moreover, the encryption of entropy-coded videos was studied in some other research works. To mention a few, one may refer to Refs. [75,76,77,78]. The impact of key entropy on the security of an image encryption scheme was studied by Ye et al. [79]. Külekci [80] investigated the security of high-entropy volumes, where the most typical sources are entropy-encoded multimedia files or compressed text sequences. Min entropy was used by Saeb [81] to reduce the size of the key search space of an encryption scheme to a value lower than that of a brute-force or birthday attack. A chaotic encryption scheme for low-entropy images was proposed by Yavuz et al. [82]. This method uses confusion and diffusion techniques to make it difficult for the adversary to perform statistical analysis on adjacent pixels, which are likely to have close values.
  - **Decryption:**
    There are few works focusing on the security of the encryption algorithm. For example, the multiple decryption problem was introduced by Domaszewicz and Vaishampayan [83] as a generalization of the problem of source coding subject to a fidelity criterion. They used entropy to evaluate the security of a multiple-channel system in this scenario.
- **Key Generation and Management Module:** Several research works have focused on the role of entropy in key generation and management. Some of these works are briefly reviewed in the following.
  The entropy of the key has been of interest to researchers as a measure of security for decades [84]. Golic and Baltatu [85] used Shannon entropy analysis to evaluate the security of their proposed biometric key generation scheme. Wang et al. [86] tried to alleviate the quantization discrepancy problem in quantization-based key generation methods using an ECQS (entropy-constrained-like quantization scheme).
  It was highlighted by Shikata [87] that the existing bounds on the key entropy for retaining information-theoretical security are not tight enough. The reason is that existing random number generators do not create truly random sequences. More realistic bounds for the key entropy were derived in this research. "Personal Entropy" was introduced by Ellison et al. [88] as a means for remembering personal passphrases based on which secret keys are generated. Personal entropy is created via asking the user several personal questions.
- **Key Agreement and Exchange Protocol:** There are a few research works focusing on the applications of entropy in key exchange protocols. Among these works, we can refer to a framework designed by Luo et al. [89] for fingerprinting key exchange protocols using their impact on high-entropy data blocks. Another example is the key transmission method presented by Boyer and Delpha [90] for MISO (multiple-input single-output) flat-fading channels. This method tries to increase relative entropy using an artificial noise in order to minimize the BER (bit error rate) for the key receiver, while keeping it close to unity (maximum) for the eavesdropper.

#### 3.1.3. Cryptographic Primitives

Several researchers have used entropy in their research works focusing on the design and implementation of cryptographic primitives, such as random number generations and hashing. Some of these works are studied below.

- **Random Number Generation Algorithm:** Entropy has been considered by researchers as a measure for randomness for decades [91]. One well-known issue with pseudo-random number generators is that the entropy of their output depends on the entropy of the seed. This issue was reported by Kim et al. [92] to exist in entropy sources of random number generators used in real-world cryptographic protocols, such as SSL (secure socket layer). Several research works have proposed methods for increasing the entropy of the seed via harvesting entropy from execution times of programs [93] or chaotic functions [94]. In recent years, entropy has been used as an objective for improving different chaos-based random number generators [95,96] as well as randomness tests for image encryption [97,98]. The criticality of entropy in research on true random number generators is due to the fact that their susceptibility to process variations as well as intrusion attacks, degrades the generated entropy. This makes it necessary to include an on-the-fly mechanism for the detection and correction of bias variations [99].
- **Hashing Functions and Algorithms:** A hash function with maximized conditional entropy was used by Lin et al. [100] as part of the solution to the ANN (approximate nearest neighbor) problem. Later on, Wang et al. [101] suggested LSH (locality sensitive hashing) as a promising solution to the ANN problem. However, they argued that in LSH, points are often mapped to poor distributions. They proposed a number of novel hash map functions based on entropy to alleviate this problem. Maximum-entropy hash functions were used in some other applications, such as packet classification [102]. The role of graph entropy in perfect hashing was studied by Newman et al. [103]. Later on, a graph entropy bound was calculated by Arikan [104] for the size of perfect hash function families. A fuzzy hash method based on quantum entropy distribution was used to construct a biometric authentication algorithm by Cao and Song [105]. Entropy measurement and improvement techniques were used by Zhang et al. [106] along with perceptual hashing for key frame extraction in content-based video retrieval. Moreover, entropy reduction on layout data combined with lossless compression and cryptographic hashing was used by Koranne et al. [107] to manage IP (intellectual property) via tracking geometrical layout from design through manufacturing and into production. Inaccessible entropy was used in the design of one-way hash functions by Haitner et al. [108]. A generator *G* is said to have inaccessible entropy if the total accessible entropy (calculated over all blocks blocks) is considerably smaller than the real entropy of *G*’s output. The possibility of designing a hash function with a hash-bit-rate equal to the conditional entropy was investigated by Li et al. [109].

#### 3.1.4. Cryptographic Hardware

Entropy measures and related concepts have played important roles in the design and implementation of cryptographic hardware, such as hardware random number generators and physically unclonable functions. In the following, we study these roles.

**Figure 3:**
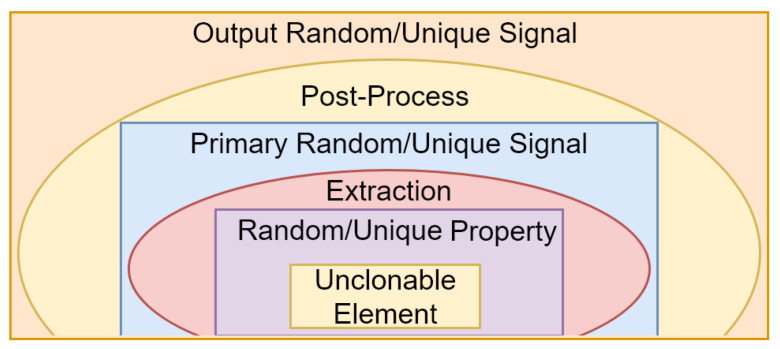
PUF architecture.

- **Hardware Random Number Generators:** The metal oxide semi-conductor (CMOS) implementation of full-entropy true random number generators was investigated by Mathew et al. [110,110]. CMOS is a fabrication process used in integrated circuits with high noise immunity and low static power consumption. Cicek et al. [111] proposed architectures for the CMOS implementation of true random number generators with dual entropy cores. In these implementations, different entropy sources, such as MRAMs [112], beta radioisotopes [113], the jitter of event propagation in self-timed rings [114] or thermal phenomena [115], were examined by researchers. Other hardware implementations depend on field programmable gate arrays (FPGAs) [116,117] or system-on-chip (SoC) devices [115]. An FPGA is a programmable semiconductor device consisting of a matrix of configurable logic blocks connected via networks of bistate connections. Furthermore, an SoC is a single integrated circuit containing (almost) all components of a computer such as a central processing unit, secondary storage, input/output ports, memory, etc. In the hardware implementation of true random number generators, objectives, such as power consumption [118], were considered by researchers.
- **Physically Unclonable Functions (PUFs):** In recent years, it was shown that some unclonable properties in some elements such as devices, waves or materials can vary randomly in different experiments or uniquely between similar elements. PUFs use these properties to create random and/or unique signals. They are used in cryptographic primitives, such as random number generation as well as message/device authentication. PUFs have been of interest to researchers in recent years [119,120]. The architecture of a PUF is shown in Figure 3.
  As shown in Figure 3, the core of a PUF is an unclonable element to which we simply refer as the element for short. The element can be a material, such as paper, carbon nanotube, etc. It can even be a wave, such as an optical or magnetic wave. However, most commonly, it is a device. It varies from sensors to microprocessors. The element along with its unique/random property (property for short) build the source of uniqueness/randomness (source for short). The property varies from eye-opening oscillation in humans to the geometry of the substrate in CMOS devices. As shown in Figure 3, an extraction circuit extracts this randomness, and (possibly) some post-processing improves the performance of the resulting signal to create the final output signal.
  Entropy analysis has appeared in several research reports focusing on the implementation of PUFs. For example, a connection between the min entropy and the randomness of PUFs was established by Gu et al. [121]. Gu et al. [122] and Schaub et al. [123] used entropy to evaluate the randomness of PUFs. Similarly, Koyily et al. [124] used entropy to evaluate the non-linearity of PUFS. Upper bounds on the entropy of some types of PUFs were calculated by Delvaux et al. [21]. Some bounds on the conditional min entropy of PUFs were presented by Wilde et al. [125]. Liu et al. [119] argued that some previously calculated upper/lower bounds on the entropy of PUFs are too loose or too conservative. They proposed a method for calculating a new bound via predicting the expectation of the point where min entropy bounds obtained from different experiments will converge. The loss of entropy in key generation using PUFs was studied by Koeberl et al. [126]. Other research works used PUFs as pumps of entropy [120].

#### 3.1.5. Modification and Use of Existing Cryptosystems

Some researchers have modified existing cryptosystems and used entropy (and related concepts) in part of their research. For example, a modified variant of the block encryption algorithm *Blowfish* was designed by Nagpal et al. [127] in order to be used in IoT (Internet of Things) platforms. The security of this variant was evaluated using entropy. ElGamal elliptic curves were used to improve the TLS (transport layer security) protocol in terms of entropy [128]. A chaotic image scrambling method based on DES was proposed by Zhang et al. [129] that uses image block entropy to select blocks for scrambling.

Entropy analysis was used in the design of an image encryption system aiming at the reduction of correlation between image blocks [130]. Moreover, entropy analysis has played roles in the design of elliptic curve point addition algorithms [131] and unconditionally secure encrypted authentication schemes [132].

### 3.2. Analysis and Evaluation

In this subsection, we first discuss entropy as a security measure. Afterwards, we review the research reports that have used entropy in the analysis of cryptographic systems, schemes, mechanisms, etc.

#### 3.2.1. Entropy as a Security Measure

Some researchers used entropy as an independent measure, and others considered it in relation with other security measures. Some effort was spent on the formal assessment of the role of entropy in the evaluation of a cryptosystem [133]. Moreover, some research reports focused on developing methods for measuring the entropy of a cryptosystem [134]. In the following, we review the related research works.

- **As an Independent Measure:** Entropy is a widely used security measure. Among the research works that have used entropy as an independent measure for evaluating cryptographic schemes, one may refer to the following. A multichannel system was introduced by Voronych et al. [135] for the purpose of structuring and transmitting entropy-manipulated encrypted signals. Schulman [136] argued that entropy makes a cryptographic pseudo-random number generator indistinguishable from a truly random number generator. He studied different ways of creating and increasing entropy. A method was introduced by Wua et al. [97] to measure the entropy of small blocks in an encrypted image. The average of the entropy over the blocks of an image was suggested as an efficient measure for evaluating the security of an image encryption scheme.
- **Relation with Other Cryptographic Measures:** In the following, we study the research works that have established connections between entropy and other security measures, such as unicity distance, malleability, guesswork, confusion, diffusion and indistinguishability.
  - **Unicity Distance:**
    The unicity distance of a cryptosystem is defined as the minimum number of ciphertext bits needed for an adversary with unlimited computational power to recover the key. The connection between entropy and unicity distance has been of interest to some researchers. For example, an entropy analysis presented by AlJabri [137] highlighted the unicity distance as an upper bound on the probability of the key being guessed by an eavesdropper.
  - **Malleability:**
    Consider a cryptosystem C and a function *f*. Let us assume that C encrypts a plaintext *p* to a ciphertext *c*, and encrypts f(p) to $C^{\prime }$. If there is a transform *g* that guarantees $g\left(c\right)=c^{\prime }$, then C is called a malleable cryptosystem with respect to the function *f*. The notion of non-malleable extractors was introduced by Dodis and Wichs [138] (inspired by the notion of malleability) for the purpose of symmetric-key cryptography from low-entropy keys. Later on, a widely believed conjecture on the distribution of prime numbers in arithmetic progressions was used by Dodis et al. [139] along with an estimate for character sums in order to build some new non-malleable extractors. Moreover, entropy analysis was used by Cohen et al. [140] to present an unconditional construction for non-malleable extractors with short seeds. Recently, some researchers worked on entropy lower bounds for non-malleable extractors [141].
  - **Guesswork:**
    There is a clear relation between entropy and guesswork. While entropy can be interpreted as the average number of guesses required by an optimal binary search attack to break a cryptosystem, guesswork is defined as the average number of guesses required in an optimal linear search attack scenario [142]. It was shown by Christiansen and Duffy [143] that if appropriately scaled, when the key is long enough, the expectation of the logarithm of the guesswork approaches the Shannon entropy of the key selection process. A similar research work studied the relation between guesswork and Rényi entropy [144]. Pliam [145] demonstrated that there cannot be any general inequality between Shannon entropy and the logarithm of the minimum search space size necessary to guarantee a certain level of guesswork. Another research reported by Malone and Sullivan [146] showed that entropy and guesswork cannot be interchangeably used in normal conditions. The LDP (large deviation principle) was used by Malone and Sullivan [147] to derive the moments of the guesswork for a source of information determined by a Markov chain. It was shown by Lundin [148] how entropy and guesswork can be simultaneously used to evaluate the security of selectively encrypted information.
  - **Confusion and Diffusion:**
    Confusion and diffusion are two properties suggested by Shannon [1] in order to make the statistical analysis of a cryptosystem as difficult as possible. Confusion states that the ciphertext is a complex function of several portions of the key, and this function cannot be simplified to an easily analyzable function. On the other hand, diffusion requires that each plaintext symbol affects several symbols in the ciphertext and each ciphertext symbol is a function of several symbols in the plaintext. This property diffuses the statistical structures of the plaintext over the symbols of ciphertext. The relation between entropy and the mentioned two properties were studied in several research works. For example, entropy was used to evaluate the security of chaotic confusion–diffusion image encryption schemes [149,150]. Moreover, Wu et al. [151] used entropy improvement techniques in combination with confusion and diffusion mechanisms in their proposed cryptographic schemes.
  - **Indistinguishability:**
    Indistinguishability states that given the ciphertext corresponding to a plaintext randomly chosen from a plaintext space with only two elements (determined by the adversary), the adversary will not be able to identify the encrypted message with a probability significantly greater than that of random guessing ($\frac{1}{2}$). Indistinguishability plays a significant role in provable security. Some research works have investigated the relation between indistinguishability and entropy. As an example, one may refer the research reported by Hayashi [152]. In this research, smoothed Rényi entropy and min entropy were used to evaluate the indistinguishability of universal hash functions. Universal hash functions have many important applications in QKD (quantum key distribution), cryptography, privacy amplification (leftover hash lemma), error-correcting codes, parallel computing, complexity theory, pseudorandomness, randomness extractors, randomized algorithms, data structures, etc. (see [153,154,155,156,157,158] and the references therein).

#### 3.2.2. Applications in Security Proof

Entropy has been used in several types of security proofs including zero-knowledge proof and random oracles. Some related works are briefly reviewed in the following.

- **Zero-Knowledge Proof:**
  Zero-knowledge proof is about proving the possession of some information by one party (the prover) to the other party (the verifier) without revealing the information itself. Zero-knowledge proofs are widely studied in cryptography. Goldreich et al. [159] further developed the notion of non-interactive statistical zero-knowledge proof introduces by De Santis et al. [160]. They used entropy measures to highlight some conditions under which every statistical zero-knowledge proof can be made non-interactive. Lovett and Zhang [161] studied some black box algorithms in order to be used in zero-knowledge proofs. These algorithms can reverse the entropy of a function. It was shown in this report that a black box function of this type incurs an exponential loss of parameters, which makes it impossible for such an algorithm to be implemented in an efficient way. A new hard problem related to lattices, named ILP (isometric lattice problem) was introduced by Crépeau and Kazmi [162], who used entropy to show that there is an efficient zero-knowledge proof for this problem.
- **Random Oracle:**
  Random oracles are widely used in security proofs in order to model perfect hash algorithms. A random oracle is a hypothetical black box that responds to each query by producing a truly random number uniformly chosen from a predefined domain. There are a few research works that use entropy-related concepts in the analysis of random oracles. For example, it was demonstrated by Muchnik and Romashchenko [163] that random oracles cannot help the extraction of mutual information.

Moreover, Imai et al. [164] worked on information-theoretic proofs, and the relations among different information-theoretic security proofs were studied by Iwamoto et al. [165].

#### 3.2.3. Applications in Adversarial Analysis

In the following, we review the role of entropy in adversarial analysis procedures, including cryptanalysis, eavesdropping, encrypted data analysis, covert channels and attacks.

- **Cryptanalysis:** Entropy measures have been frequently used in research works focusing on cryptanalysis [166]. In particular, chaotic image encryption methods were cryptanalyzed using entropy calculations [167]. Moreover, some researchers used different methods for the cryptanalysis of chaotic image encryption schemes that use entropy improvement techniques [168].
- **Eavesdropping:** Measures of mutual information in quantum key distribution and their applications in eavesdropping were investigated by Rastegin [30].
- **Encrypted Data Analysis:** The analysis of encrypted data is another relevant area of application for entropy. For example, entropy analysis was used for identifying encrypted malware [169], detecting encrypted executable files [170], and correcting noisy encrypted images [171]. Moreover, some researchers focused on entropy analysis of encrypted strings [172].
- **Covert Channel:** Entropy has played role in research on adversarial analysis of cryptosystems via covert channels. For example, entropy was used by Chen et al. [173] to analyze the capacity of a covert channel as well as the factors affecting it.
- **Attacks:** Entropy analysis was used as part of several kinds of attack scenarios. To mention a few, we can refer to the following.
  - **CPA (Chosen Plaintext Attack):**
    Kiltz et al. [174] used entropy measures in their analysis of instantiability of RSA and optimal asymmetric encryption padding (OAEP) under a chosen plaintext attack. OAEP is a padding scheme proposed by Bellare and Rogaway [175], which is often used along with RSA encryption. In another research reported by Bard [176], entropy was used in a CPA against SSL. Moreover, Bard [177] tested several modes of operation for resistance against a blockwise adaptive chosen plaintext attack.
  - **CCA (Chosen Ciphertext Attack):**
    Like the case of chosen plain text attack, entropy analysis has played role in chosen ciphertext attack adversarial analysis. For example, a public-key cryptosystem featuring resistance against CCA was introduced by Zhao et al. [178]. Entropy assessment was used in order to prove the security of this cryptosystem against after-the-fact leakage without non-interactive zero-knowledge proof. Similarly, Sun et al. [179] presented a CCA-secure identity-based encryption system and used entropy to show its resistance against key leakage attacks. Another research study on CCA-resistant and leakage-resistant cryptosystems was reported by Zhou et al. [180] in which entropy was used in the security proof.
  - **Side Channel Attack:**
    Mutual information measure is frequently used in side channel attacks. The reason is that mutual information is capable of detecting any kind of statistical dependency, and many side channel analysis scenarios depend on a linear correlation coefficient as a wrong-key distinguisher [181]. Moreover, some research works have used entropy analyses to make cryptosystems more secure against side channel attacks. For example, a method for decreasing the entropy of the information leaked from side channels was introduced by Dhavlle et al. [182]. As another example, an information-theoretical model for side channel attacks was derived by Köpf and Basin [183]. The impact of the entropy of the masks in masking-based countermeasures against side channel attacks was studied by Nassar et al. [184]. This study shows that while these countermeasures are usually studied with the maximal possible entropy for the masks, some particular mask subsets may leak remarkably more as the entropy increases.
  - **Replay Attack:**
    Entropy analysis has been used in the detection of replay attacks. As an example, we can mention the research reported by Liu et al. [185], wherein a novel feature based on spectral entropy was introduced for detecting replay attacks.
  - **Key Negotiation Attack:**
    It was shown by Liu et al. [186] and Antonioli et al. [187] that an attacker can manipulate the key entropy negotiation protocols used by Bluetooth and Bluetooth low energy and notably reduce the encryption key space.
  - **Backdoor Attack:**
    As an example of the applications of entropy in backdoor attacks, we can mention the research reported by Young and Yung [188]. They argued that some backdoor attacks, such as *Monkey*, require the attacker to obtain a large number of ciphertext blocks all encrypted by the same symmetric key, each containing one known plaintext bit. They proposed a new backdoor that eliminates the need for known plaintext while leaking a bound on the plaintext entropy to the reverse engineer.
  - **Dictionary Attack:**
    Some researchers have worked on the role of entropy in dictionary attacks. For example, it was shown by Nam et al. [189] that low-entropy keys make some PAKE (password-authenticated key exchange) protocols, such as the one presented by Abdalla and Pointcheval [190], vulnerable to dictionary attacks.
  - **Algebraic Attack:**
    In addition to dictionary attacks, low-entropy keys make cryptosystems vulnerable to algebraic attacks. For example, the complexity of finding low-entropy keys using SAT (Boolean satisfactory problem) solvers was studied by Hromada et al. [191].
  - **Collision Attack:**
    It was demonstrated by Rock [192] that replacing random permutations by random functions for the update of a stream cipher causes entropy loss, which makes the cipher vulnerable to collision attacks.
  - **Correlation Attack:**
    Wiemers and Klein [193] argued that the *correlation-enhanced power analysis collision attack* against AES proposed by Moradi et al. [194] usually yields a set of keys (instead of one) due to noise-related problems. To alleviate this problem, they proposed a practical search algorithm based on a theoretical analysis on how to quantify the remaining entropy.

#### 3.2.4. Analysis of Well-Known Cryptographic Schemes

In the following, we review the applications of entropy in the analysis and evaluation of well-known traditional or modern cryptographic schemes, algorithms, systems and mechanisms.

- **Analysis of Traditional Cryptosystems:** Several researchers have made use of entropy analysis in their evaluations of well-known traditional cryptographic schemes. For example, Bivium [195], RSA [196], AES [197], DES [198] and SOBER-t [199] were subject to entropy analysis by different researchers.
- **Analysis of Emerging Cryptographic Paradigms:**
  In addition to traditional cryptographic schemes, entropy has been used in research on cutting edge cryptographic schemes and paradigms, such as quantum cryptography, homomorphic encryption, white-box cryptography and attribute-based encryption. Some related research works are briefly reviewed in the following.
  - **Quantum Cryptography:**
    Entropy was used by Bienfang et al. [200] and Bienfang et al. [201] in order to evaluate OTP video stream encryption that use quantum-generated secret keys. Arnon-Friedman et al. [202] used entropy to analyze the security of a device-independent quantum cryptography scheme. Moreover, entropy was used in several research works for the purpose of evaluating QKD (quantum key distribution) protocols [203,204].
  - **Attribute-Based Encryption:**
    A technique aimed at increasing the entropy available for proving the security of dual system encryption schemes under decisional linear assumption was presented by Kowalczyk and Lewko [205]. They showed the efficiency of their method in an attribute-based encryption scheme as a case study.

#### 3.2.5. Analysis of Cryptographic Problems and Functions

Entropy was used in the analysis of several cryptographic functions. One-way functions (OWF) have many applications in cryptography [206]. Some of these functions were analyzed using different entropy notions. For instance, two computational notions of entropy, namely, “next-block pseudoentropy” and “inaccessible entropy” were used by Haitner and Vadhan [207] for analyzing and comparing some one-way functions.

### 3.3. Application

Cryptosystems and cryptographic schemes designed, implemented and evaluated using entropy, were used in a variety of platforms, environments, applications and computing paradigms, among which we can mention fog-based IoTs [208]. In fog-based IoT, a nearby light-weight middleware is used to bridge the gap between IoT deices and the far-away cloud. This helps provide the required support and communication between devices, sensors, receptors on one side and and the servers on the other side. Even entropy-as-a-service was proposed by some researchers [209]. However, among these environments, we will discuss blockchain and cryptocurrencies in more detail because of their importance in the modern world.

The possibility of using the inherent unpredictability of blockchains and especially Bitcoin as a source of randomness was investigated by Pierrot and Wesolowsk [210]. They demonstrated that random numbers generated by this method are malleable in the sense that an adversary will be able to manipulate them, even with limited computational power and financial budget. Some indicators for evaluating public blockchains were introduced by Yong and Peiwu [211]. They used entropy for ranking the indicators. Wu et al. [212] argued that while decentralization is a critical selling point of most public blockchain platforms, most research works on this property fail to quantify it and perform calculations on it. They presented a method based on entropy for quantification of the degree of decentralization in blockchains.

## 4. Entropy and Other Cryptographic Areas

There are several areas related to cryptography in the field of information security. In this subsection, we briefly review some of these areas along with the role of entropy in the research on each area.

### 4.1. Obfuscation

Obfuscation is the act of making a source of information difficult to understand without the use of a key or an external source of randomness (entropy). For example, in software development, vague names can be assigned to routines or variables in order to obfuscate the code. Entropy was used by Giacobazzi and Toppan [213] to model the uncertainty created by obfuscation and its impact on software security.

### 4.2. Message Authentication Codes

Cheng et al. [214] argued that entropy attacks can adversely affect the throughput of P2P live streaming systems that use network coding (entropy attacks are similar to pollution attacks, wherein the attacker fabricates and transmits polluted packets, avoiding linear combinations of previously sent original packets, but in this case, the attacker tries non-innovative packets containing information already known by the system). They proposed a message authentication system based on symmetric key homomorphic encryption to facilitate the detection of entropy attacks in these systems.

Entropy loss during generic distinguishing-H and state-recovery attacks against hash-based message authentication codes (such as HMAC and NMAC) above the birthday bound was studied by Leurent et al. [215]. Generic distinguishing-H attack aims at distinguishing between different MAC algorithms (e.g., between HMAC and NMAC) to pave the way for more complex attacks. State recovery attack against a cryptographic primitive, as suggested by the name, tries to recover the hidden internal state of an algorithm run by the primitive. Leurent et al. showed that trying to detect collisions by repetitive invocations of a random number generating function does not give a random collision, and this method makes some particular collision patterns much more likely to be detected than others.

### 4.3. Cryptography-Based Privacy

Entropy has played a significant role in several research works in the area of privacy. For example, Rényi entropy and inf-spectral entropy (defined by Bowen and Datta [216]) were used by Watanabe and Hayashi [217] for the purpose of non-asymptotic analysis of privacy amplification. They compared two existing bounds for the *“privacy amplification"* problem and used the mentioned entropy measures to present an interpolation-based method for achieving a new bound for this problem.

Both traditional and differential privacy were studied by Yao [218] from the perspective of α-mutual information (introduced in the same research report). They proposed a unified framework for analyzing the relations between statistical security and mutual information security for a number of different privacy schemes. A mechanism for achieving differential privacy in linear distributed control systems was presented by Wang et al. [219]. This mechanism is based on adding Laplace noise to the shared information depending on the sensitivity of the control system to the private data. They calculated a lower-bound for the entropy of any unbiased estimator of the private data from any noise-adding mechanism giving differential privacy.

#### 4.3.1. Steganography and Steganalysis

Perfectly secure steganography takes great advantage of entropy analysis [220]. In this kind of steganography, the covertext keeps its statistical distribution despite the added hidden text. This makes the stegotext statistically indistinguishable from the covertext. The entropy of image blocks was used by Hu et al. [221] to define a novel distortion measure for steganographic schemes based on frequency domain transformations such as DCT (discrete cosine transform). A framework for blind decoding of image steganography was presented by Kim et al. [222] that is based on measuring the local entropy distributions of decoded images. It was shown in this report that the local entropy distributions of incorrectly decoded images are different from those of normal ones, and the reason is the abnormal structures in erroneously decoded images. Zheng and Cox [223] argued that the existence of a correlation between the cover image and the payload reduces the number of bits needed to hide a given message. They reasoned that this reduction is due to the fact that the correlation decreases the conditional entropy of the message given the cover. A variable-rate image steganography method was proposed by Roy and Changder [224] that tunes the data embedding rate in each image block based on the block entropy. In addition to steganography, some researchers investigated the use of entropy in steganalysis [225].

### 4.4. User/Device Authentication

Authentication is another cryptography-related area in which researchers used entropy for design and evaluation purposes. For example, entropy has played roles in some research works focusing on the trust evaluation of biometric user authentication systems [226]. As another example, one can mention the application of entropy in the design of image encryption, authentication and compression systems [227]. A comparative study of the applications of different entropy measures in the authentication of EEG (electroencephalogram) signals was presented by Mu et al. [228].

### 4.5. Digital Signature

Researchers have frequently used entropy in their research on digital signature. As an example, we can refer to the research reported by Atighehchi and Barbier [229], which focused on the problem of publicly authenticating low-entropy data, or data with slight variations over time. Moreover, entropy and related concepts were used in research reports focusing on proxy signature, blind signature and signcryption. For example, entropy was used by Verma and Dhir [230] as a performance measure for threshold proxy signature schemes based on RSA encryption. Rückert [231] used min entropy to evaluate lattice-based blind signatures. Dent et al. [232] presented formal security models for deterministic signcryption schemes with high/low-entropy input messages, and proved encrypt-and-sign to be a secure scheme for high-entropy messages.

### 4.6. Secret Sharing

Secret sharing is the science of dividing a secret into shares and distributing them among a number of participants in a way that the secret can be retrieved only when a predetermined minimum number of shares are collected from participants. For example, in Shamir’s (k,n) secret sharing scheme, the secret *s* is distributed among *n* participants in a way that *k* of the participants can reconstruct *s*. The procedure is as follows. The dealer (the party who owns the secret) chooses k−1 random numbers $a_{1},a_{2},\ldots ,a_{k-1}$, and builds a polynomial $S\left(x\right)=a_{0}+\sum_{i=1}^{k-1}a_{i}x^{i}$, where $a_{0}=s$. Afterwards, the dealer calculates $S_{i}=S\left(i\right)$ for all i∈{1,2,…,n} and privately sends them to *n* participants $P_{1},P_{2},\ldots ,P_{n}$. The polynomial S(x) and consequently the secret *s* can be reconstructed using any subset $S^{\prime }$ of $S=\{S_{1},S_{2},\ldots ,S_{n}\}$ via an interpolation method, such as Lagrange interpolation, provided that $|S^{\prime }|\geq k$.

There are some secret recovery methods that eliminate the need for remembering the secret shares via making it possible to recover each share by asking the owning participant a number of personal questions [88]. Moreover, entropy was used to model and design more generalized secret sharing systems wherein more than one secret is shared, and each secret can be reconstructed only by a qualified set of participants [233].

## 5. Concluding Remarks

**Figure 4:**
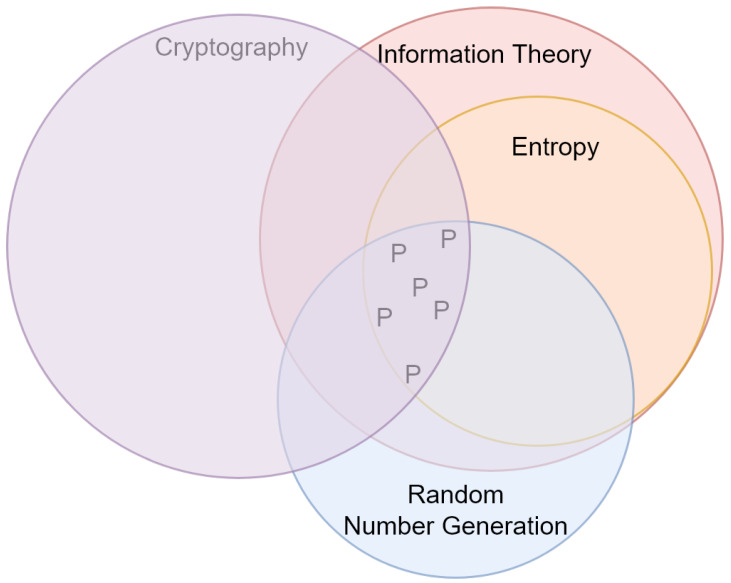
Current trends in the application of entropy in cryptography. Circles represent areas of research and the symbol ‘P’ denotes the hot topic ‘*applications of entropy in truly random number generation*’.

In the previous sections, we showed the importance of entropy and information theory in research on cryptography. In particular, modern cryptography highly depends on randomness extraction and truly random number generation [234]. PUFs are good examples of devices designed for these purposes (see Section 3.1.4). Furthermore, entropy plays a significant role in recent research on truly random number generation [96,99]. Future researchers may consider reviewing the role of other concepts, such as chaos and complexity, in cryptography. This trend is shown in Figure 4. In this figure, every circle represents the area mentioned, and the area highlighted by ‘P’ symbols shows the hot topic *‘applications of entropy in truly random number generation’*. This is the research topic which is identified as a dominating trend.

## Data Availability

Not applicable.
